# Investigating Evolutionary Conservation of Dendritic Cell Subset Identity and Functions

**DOI:** 10.3389/fimmu.2015.00260

**Published:** 2015-06-02

**Authors:** Thien-Phong Vu Manh, Nicolas Bertho, Anne Hosmalin, Isabelle Schwartz-Cornil, Marc Dalod

**Affiliations:** ^1^UM2, Centre d’Immunologie de Marseille-Luminy (CIML), Aix-Marseille University, Marseille, France; ^2^U1104, Institut National de la Santé et de la Recherche Médicale (INSERM), Marseille, France; ^3^UMR7280, Centre National de la Recherche Scientifique (CNRS), Marseille, France; ^4^Virologie et Immunologie Moléculaires UR892, Institut National de la Recherche Agronomique, Jouy-en-Josas, France; ^5^INSERM U1016, Institut Cochin, Paris, France; ^6^CNRS UMR8104, Paris, France; ^7^Université Paris Descartes, Paris, France; ^8^Assistance Publique-Hôpitaux de Paris (AP-HP), Hôpital Cochin, Paris, France

**Keywords:** mononuclear phagocytes, comparative genomics, human, non-human primates, mouse, pig, sheep, chicken

## Abstract

Dendritic cells (DCs) were initially defined as mononuclear phagocytes with a dendritic morphology and an exquisite efficiency for naïve T-cell activation. DC encompass several subsets initially identified by their expression of specific cell surface molecules and later shown to excel in distinct functions and to develop under the instruction of different transcription factors or cytokines. Very few cell surface molecules are expressed in a specific manner on any immune cell type. Hence, to identify cell types, the sole use of a small number of cell surface markers in classical flow cytometry can be deceiving. Moreover, the markers currently used to define mononuclear phagocyte subsets vary depending on the tissue and animal species studied and even between laboratories. This has led to confusion in the definition of DC subset identity and in their attribution of specific functions. There is a strong need to identify a rigorous and consensus way to define mononuclear phagocyte subsets, with precise guidelines potentially applicable throughout tissues and species. We will discuss the advantages, drawbacks, and complementarities of different methodologies: cell surface phenotyping, ontogeny, functional characterization, and molecular profiling. We will advocate that gene expression profiling is a very rigorous, largely unbiased and accessible method to define the identity of mononuclear phagocyte subsets, which strengthens and refines surface phenotyping. It is uniquely powerful to yield new, experimentally testable, hypotheses on the ontogeny or functions of mononuclear phagocyte subsets, their molecular regulation, and their evolutionary conservation. We propose defining cell populations based on a combination of cell surface phenotyping, expression analysis of hallmark genes, and robust functional assays, in order to reach a consensus and integrate faster the huge but scattered knowledge accumulated by different laboratories on different cell types, organs, and species.

## Introduction

**Figure 1:**
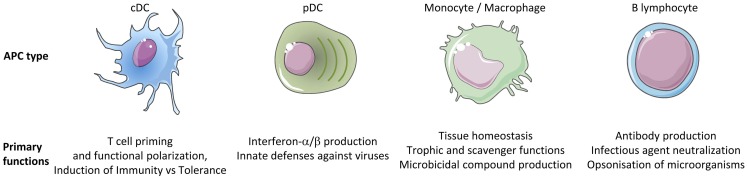
**Different types of APCs are specialized in distinct primary functions**. cDC are uniquely efficient for the priming and functional polarization of T cells. Although other APCs also contribute to this process, this does not represent their primary functions. Hence, cDC play a central and non-redundant role in the orchestration of adaptive immunity.

**Figure 2:**
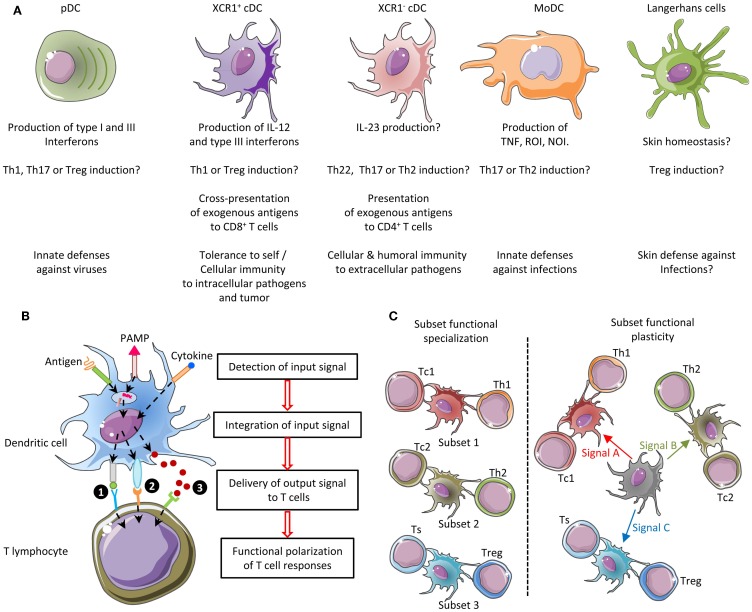
**Combined functional specialization and plasticity of DC subsets allows mounting different types of adaptive immune responses adapted to the various natures of the threats to be faced**. **(A)** Five DC subsets can be defined in mice based in part on their functional specialization: pDC, XCR1^+^ cDC, XCR1^−^ cDC, MoDC, and Langerhans cells. Certain DC subsets are more efficient than others to exert a specific function, because they are intrinsically genetically built to activate this function faster and in more diverse settings. **(B)** The function of each DC subset is relatively plastic. Three types of output signals are delivered by DC to T cells and instruct their functional polarization: (1) ligands for the T-cell receptor (antigenic peptides presented in association with MHC molecules), (2) co-stimulation, and (3) cytokines. Co-stimulation and cytokine signals can be either activating (e.g., CD86 and IL-12, respectively) or inhibitory (e.g., PD-L1 and IL-10, respectively). Different cytokines induce distinct types of helper T-cell responses. For example, IL-12 primarily promotes Th1, IL-4 promotes Th2, and IL-23 promotes Th17. Each DC subset can sense a specific array of microbial or danger signals. Integration of the particular combination of input signals received by the DC in a given pathophysiological context determines the precise type of maturation ensuing and hence the combination of output signals delivered to T cells. As a result, different DC subsets can exert similar or complementary functions depending on the physiopathological context. **(C)** The combination of functional specialization and plasticity of subsets allows DC responses to be highly flexible and thus to react rapidly to different threats by coupling the type of danger sensed to the most appropriate type of immune response to induce for protection. However, this flexibility can lead to confusion if attempting to define DC subsets only on functional specialization. NOI, nitric oxide intermediates; ROI, radical oxygen intermediates; Th, T helper cell; Tc, cytotoxic T cell; Treg, regulatory T cell; Ts, T suppressor cell.

The immune system includes a large variety of myeloid and lymphoid cell types which develop through distinct ontogenic pathways, express specific phenotypes, and exert specialized functions. The mononuclear phagocytes form a complex group of myeloid cells that encompass three major cell types, i.e., monocytes, macrophages, and dendritic cells (DC), together with their proximal progenitors. These three cell types contribute to maintain host integrity by shaping the innate and adaptive immune defense, a generic function related to their common phagocytic properties and their capacity to present antigen to T cells. These functions are also shared by other types of professional antigen-presenting cells (APCs), in particular B lymphocytes. However, different types of APCs are primarily devoted to distinct functions (Figure 1). B cells produce antibodies. Monocytes patrol the organism for the detection of pathogens and dominantly display inflammatory and oxidative stress response. Macrophages mainly perform microbicidal, scavenging, and tissue trophic/maintenance functions. DC are uniquely efficient for antigen-specific activation of naïve T lymphocytes, a process called T-cell priming. Indeed, DC were initially defined by their dendritic morphology and their exquisite capacity for T-cell priming. DC include two main cell types, the plasmacytoid DC (pDC) that are expert in type I interferon synthesis upon viral stimulation and the conventional DC (cDC) that are specialized in antigen capture, processing, and presentation for T-cell priming. Two cDC subsets can be distinguished based on a further segregation of functions. XCR1^+^ cDC1 are particularly efficient in CD8^+^ T-cell activation and cross-presentation, at least in mice. XCR1^−^ cDC2 are most efficient for T helper cell priming, in particular polarization toward Th2 or Th17, and for the promotion of humoral immunity. Importantly, an additional layer of complexity is generated by the plasticity of the different mononuclear cell types, which display modified phenotypes and functions contingent to the anatomical microenvironment where they reside or when exposed to pathogens or inflammation. For instance, monocytes adopt a dendritic morphology and antigen-presentation functions in inflammatory settings (1–3) as well as when located in the dermis (4–6), leading to their designation as monocyte-derived DC (MoDC). Langerhans cells, long considered to be DC due to their morphology and antigen-presentation function, are now known as a type of tissue macrophages (7–13). More generally, the gene expression programs, phenotypes, and functional properties of macrophages are strongly influenced by their tissue of residence. Finally, not only XCR1^+^ cDC but also other DC subsets including pDC and XCR1^−^ cDC can also efficiently cross-present antigens to CD8^+^ T cells when appropriately stimulated (14–22). Thus, the plasticity of the mononuclear phagocyte responses superimposes onto the segregation of phenotypes and functions attributed to subsets (Figure 2), which can lead to confusion in the definition of the different cell types if only based on functional assays. Hence, morphologic, phenotypic, and functional criteria are not sufficient to rigorously define mononuclear phagocyte subsets, and to properly discriminate what are distinct cell types as opposed to different developmental or activation states of a given cell type. Complementary or robust alternative criteria are needed to rigorously define the identity of the mononuclear phagocyte subsets.

Mononuclear phagocyte subsets were recently shown to develop from distinct progenitors and/or under the instruction of different transcription factors or cytokines. cDC and pDC derive from a dedicated bone marrow precursor, the common DC progenitor, with a differentiation potential strictly restricted to this hematopoietic lineage. pDC and cDC homeostasis exquisitely depends on the growth factor FLT3-L. pDC development strictly depends on the transcription factors TCF4 (E2-2) and SPIB both in mouse and human, XCR1^+^ cDC development on the master transcription factor IRF8 at least in mice, and XCR1^−^ cDC development on IRF4. Macrophages derive from a monocytic precursor, either of embryonic origin as in the case of Langerhans cells and microglia, or at least in part from circulating blood monocytes as in the case of gut macrophages. Egress of classical monocytes from the bone marrow into the blood strictly depends on the chemokine receptor CCR2. As a consequence, in competitive mixed bone marrow reconstitution experiments in mice, all cell types derived from circulating blood monocytes are primarily reconstituted from wild-type cells and not from CCR2-deficient cells. Hence, it has been proposed that the study of their developmental pathway, in other words ontogeny, was the best way to classify mononuclear phagocyte cell types, at least in the mouse model where the knowledge in DC subset properties is also the most advanced. Indeed, in this model, genetically modified animals unambiguously permit to track the development of cell types and to dissect their phenotypes and functions, in different contexts *in vivo*. However, the identity and functions of the different mononuclear phagocyte subsets need to be established outside of the mouse model, in animal species where ontogenic studies cannot be easily conducted, in order to accelerate translation of our advanced knowledge on the functioning of the mouse immune system toward clinical and/or economical applications to sustain global human health. Very promising vaccine and immunomodulatory strategies have been developed in mouse models based on DC subset targeting (23–35). The translation of these strategies to human and other species has not yet reached the expected success, likely due to insufficient knowledge in the identity and function of homologous DC subsets across species. This knowledge is needed in biomedical model species, primarily in non-human primates, and also in alternative models such as pigs that share physiological and anatomical similarities with humans – for instance skin and lung structural properties – and that present sensitivity to human pathogens of great importance for public health such as influenza. In addition, this knowledge is needed for companion and sport animals, and for animals of the agro-economy, such as ruminants, pigs, poultry, and fishes, with the goal to improve vaccination strategies against pathogens responsible for major economic losses, to decrease antibiotic use and to ameliorate animal welfare. These species, as well as wild animals, are also targets or reservoirs for major zoonotic pathogens whose control could thus benefit from new vaccine strategies targeting DC subsets in these animal species. This raises the question how to best define DC subset identity and functions in a way that can be extrapolated from mouse to human and other species, for clinical applications as well as for a better understanding of the evolution of the immune system.

## Different Methodologies to Define the Identity of Immune Cell Types, with Their Advantages and Drawbacks

Several methodologies have been proposed to define cell types. They include cell surface phenotyping and morphology, ontogeny, functional characterization, molecular profiling at population level, and molecular profiling at single cell level. We will discuss the specific drawbacks and advantages of each of these approaches (Table 1).

**Table 1:** **Different methodologies to define DC subsets with their advantages and drawbacks^a^**.

	Methodology				
	Cell surface phenotyping	Ontogeny	Functional characterization	Molecular profiling	
				At the population level	At the single cell level
Dependency on cell surface phenotyping	Not applicable	Yes but **methodology allows assessing the risk of cell type cross-contamination**	Yes, risk of bias	Yes, risk of bias	**No**
			Data quality heavily depends on rigor of the cell surface phenotyping procedure used to identify cell types	Data quality heavily depends on rigor of the cell surface phenotyping procedure used to identify cell types. ***A posteriori*** **analyses can allow rigorously assessing the risk of cell type cross-contamination**	***Ab initio*** **identification of cell types without use of prior knowledge on their identity**

Experimental feasibility	**Good**	Difficult for most species except mouse	Depends on the species studied and the functions tested	**Good** Needs comparison with sister cell types and potential contaminants	Challenging both for data generation and data analysis. Commercial solutions exist for data generation but are expensive
					Needs to balance cost and sequencing depth. Data analysis still in a large part dependent upon knowledge from molecular profiling at the population level

Protocol standardization	**Achievable** **soon** but currently limited. Currently used markers defined fortuitously/empirically, generally unrelated to cell biology, and different between tissues, species, and laboratories	Difficult	Difficult	**Good**	Should happen upon technology maturation and democratization
			The most subject to variations. Multiplicity of protocols depending on the functions tested, the tissues used and the species studied including its genetics, and even on the laboratories	**Routine technology for data generation** **Democratization of bioinformatics analyses**	

Frequency of use	**Most frequent**	Mostly by specialists	**Frequent**	**Increasing frequency**	Very rare but high potential
			Depending on the species studied and the functions tested		

Advancement of knowledge	The less informative	**Generally dichotomic information allowing relatively easy classification. Relevant to cell biology**	**Yes**	**Yes**	**Yes**
			**The most relevant for clinical and veterinary applications**	**Generation of novel hypotheses on the ontogeny or functions of cells and their molecular regulation. Identification of conserved and biologically relevant cell surface markers. Identification of candidate molecular targets to manipulate cell functions**	**Same advantages as molecular profiling at the population level**.
					**In addition**,
					**i) unbiased identification of cell types and associated transcriptomic signatures**,
					**ii) strong potential for identification of new cell types, iii) evaluation of intra-cell type heterogeneity, and**
					**iv) rigorous identification of cellular modules constituted of genes co-expressed in single cells and contributing to the same biological function**

*^a^Advantages are indicated in bold font and drawbacks in plain font*.

### Cell surface phenotyping and morphology

Cell surface phenotyping generally is a mandatory first step for all other proposed methodologies aiming at defining DC subsets. It may be skipped only for particular experiments of molecular profiling at single cell level and perhaps for functional tests based on validated protocols for specific depletion of the targeted cell subset *in vivo*. Indeed, phenotypic characterization/identification of DC subsets is necessary either to purify them for morphological analysis, functional assays, or molecular profiling, or to compare their characteristics in tissues or bulk cell suspensions (expression of lineage reporters in cell fate mapping experiments, anatomical location, maturation status, cytokine production, interactions with T cells…). Phenotypic characterization through cell surface phenotyping by flow cytometry is the method of DC subset identification the easiest to perform and the most frequently used. No single cell surface marker has been found to be sufficient for identification of a given DC subset, except for XCR1 expression on mouse and human XCR1^+^ cDC (18, 36–42) and maybe BDCA2 or LILRA4 expression on human pDC (43–46). Thus, to rigorously identify any given DC subset in any species with a limited risk of contamination by another cell type, most of the time complex combinations of multiple markers are required, often including the use of exclusion marker to ensure lack of contamination of the cell population targeted by other cell types sharing with it many positive markers. For example, the CD8α^+^ subset of mouse pDC can heavily contaminate mouse lymphoid organ-resident XCR1^+^ cDC when defined phenotypically as Lineage^−^ CD11c^+^CD8α^+^ (47–49). This problem can be solved by exclusion of SiglecH^+^ or CCR9^+^ cells or by using XCR1 as a positive marker. Similarly, other cells including MoDC or activated CD1c (BDCA1)^+^ XCR1^−^ cDC can heavily contaminate human XCR1^+^ cDC when defined phenotypically as Lineage^−^ HLA-DR^+^CD141 (BDCA3)^+^ (41, 50, 51). This problem can be solved by using CADM1 or XCR1 as additional positive markers (41, 52). Rigorous phenotypic identification of XCR1^−^ cDC (mouse CD11b^+^ cDC and human CD1c^+^ cDC) can be much more challenging, since these cells can be difficult to discriminate from MoDC, in particular under inflammation settings (53, 54). Identification of DC based on oligoparameter phenotyping is even more at risk of inaccuracy in other species, due to the limited panel of available antibodies directed to surface markers and to the poor knowledge in surface marker expression selectivity in non-DC cell types. However, major advances have recently been made to refine strategies for DC subset identification by cell surface phenotyping, in part based on novel knowledge gained through ontogeny and molecular profiling studies as will be discussed below. Hence, protocols for DC subset identification by cell surface phenotyping might soon become standardized, at least in mouse and human. This would allow better comparison of data across laboratories and limit the risk of use of inappropriate protocols leading to improper data interpretation. Special attention should be given to enzymatic dissociation that can strongly modify cell surface marker detection. Ideally, universal phenotyping protocols could be designed, allowing to considerably simplify the current nomenclatures for DC subsets by using the same name and similar marker combinations to identify homologous cell types irrespective of their tissues and species of origin (55–57). Moreover, the markers used to define and name DC subsets could be chosen based on their relevance to the biology of these cells, contrary to the current situation where the markers used were discovered fortuitously/empirically and may not be linked to the biology of the eponymous cells, as is the case for CD8α and CD141 for mouse and human XCR1^+^ cDC, respectively. However, when identifying a potentially new subset of DC or studying in a novel context a potentially known DC subset, a number of precautions need to be taken for data interpretation, including confirmation of conclusions by complementary methods such as ontogeny, functional, or molecular profiling studies.

### Ontogeny

Ontogeny studies in mice, in particular studies on the dependence of DC subset development on transcription factors, have been instrumental in identifying the homologies between lymphoid tissue-resident CD8α^+^ cDC and the CD103^+^CD11b^−^ cDC present in non-lymphoid tissues and migrating into the draining lymph nodes once activated (58). These studies, together with gene expression profiling analyses (9, 40), ultimately allowed grouping mouse CD8α^+^ cDC and CD103^+^CD11b^−^ cDC together under the umbrella of the XCR1^+^ cDC subset (38, 40, 59, 60). The recent discrimination of mouse CD11b^+^ cDC from MoDC has also been largely based on the analysis of the role of specific chemokine or growth factor receptors on cell type development *in vivo*, namely CCR2 dependence as a characteristic of monocytic origin and FLT3 dependence as a proof of cDC identity (2, 3, 6, 61). In addition, mouse CD11b^+^ cDC development was shown to selectively depend on the IRF4 transcription factor (62, 63). Moreover, the establishment of the concept that mouse *bona fide* DC constitute a separate hematopoietic lineage, and the discrimination between mouse CD11b^+^ cDC and MoDC, were confirmed using mutant animals allowing to track natural precursor–progeny relationships *in vivo* through irreversible fluorescent tagging of all daughter cells of a given type of hematopoietic progenitor, based on Cre-mediated conditional activation of a floxed reporter gene under the control of the constitutive Rosa26 promoter, an experimental strategy-coined fate mapping (64). Based on the important contribution of ontogenic studies for rigorous delineation of the identity of mouse DC subsets and of their lineage relationships, it has been proposed to use ontogeny as a primary methodology for the classification of mononuclear cell subsets in all species (57). Recent methodological progress has now made rigorous ontogenic studies applicable to human DC subsets, by using surrogate models of DC development from human CD34^+^ hematopoietic progenitors, either *in vitro* (41, 65, 66) or *in vivo* in alymphoid mice (66–68). Such approaches have allowed demonstrating remarkable similarities in the ontogeny of mouse and human DC subsets. For example, knock-down experiments performed by transducing human CD34^+^ hematopoietic progenitors with shRNA-expressing lentiviral vectors allowed to show that human pDC development critically depends on the transcription factor SPIB including *in vivo* in humanized mice (67), and that human XCR1^+^ cDC development depends on the transcription factor BATF3 *in vitro* but not *in vivo* in humanized mice (68). Moreover, the pathway for the development of human pDC, XCR1^+^ cDC, and XCR1^−^ cDC was very recently demonstrated to be similar to that described for mouse DC subsets, with the identification of the human homologs to the mouse common DC progenitor and pre-cDC (66, 69). The role of candidate genes susceptible to affect DC development can even be assessed *in vivo* in humans in the rare cases where patients have been identified with primary immune deficiencies resulting from natural mutations in such genes (70). Strategies are being developed to actively search for human primary immunodeficiencies affecting DC development as experiments of nature allowing deciphering the molecular mechanisms regulating this biological process (71). However, ontogenic studies will often not be applicable in human for rigorous assessment of the identity of DC subsets, for example when studying a potentially known DC subset in a novel physiopathological context, including characterization of the DC subsets present in steady-state non-lymphoid tissues (50) or infiltrating tumors and their draining lymph nodes (72, 73) or isolated from infected/inflamed tissues. In addition, rigorous ontogenic studies will be very difficult to perform in many species, because (i) precursor/progeny relationships remain very difficult to evaluate *in vivo* through cell fate mapping or cell transfer experiments, (ii) *in vivo* analysis of cell subset development dependence on growth factors or transcription factors cannot be reasonably done due to operational and/or financial reasons, and (iii) *in vitro* models of *bona fide* DC development are currently lacking (74). Hence, the use of other methodologies will be necessary to prove DC subset identity in these various conditions.

### Functional characterization

Ideally, cell types should be defined based on the array of functions they can exert, because this definition links identity to function and is hence the most relevant to understand the functioning of the immune system and to harness the biology of DC subsets for improving health care of humans and of other species. In addition, cell type definitions based on their functional specialization could be the most universal across tissues and species. However, functional assays are often the hardest to perform experimentally and can be the most subject to variations depending on assays and experimental conditions. This is especially the case for assays aiming at comparing the ability of different DC subsets to activate T cells. If one aims at precisely comparing the cell-intrinsic ability of different DC subsets to process and present antigens, a number of potentially confounding factors must be taken into account to design the experiment in order to reduce the risk of inappropriate interpretation of results. Adequate steps must be taken to preserve the viability of DC subsets and control for it. This implies adding to each isolated DC subset the appropriate cytokines or growth factors necessary for their survival, for example GM-CSF for cDC and IL-3 for human pDC. For instance, sorted XCR1^+^ cDC show a lower *ex vivo* survival as compared to XCR1^−^ cDC in mice and sheep (75, 76). Sorting of DC subset by positive selections may affect DC subset responses due to antibody-mediated receptor stimulation (43, 77–79). This also implies including a positive control consisting in DC subsets pulsed with optimal epitopic peptides, to assess on antigen-specific T-cell priming by DC the impact of other factors than DC subset-intrinsic differences in antigen processing and presentation, not only differences in DC subset viability but also in delivery of co-stimulation or cytokine signals. In this regard, for a fair comparison between DC subsets, they should each be matured by stimulation with an appropriate adjuvant. PolyI:C is much more efficient than LPS for the activation of human XCR1^+^ DC while it is the reverse for the activation of human MoDC. TLR7 or TLR9 ligands, but not TLR3 or TLR8 ligands, are potent activators of human pDC. Another layer of complexity is due to fundamental differences in the design of experiments in different species. While the gold standard for antigen processing and presentation assays in mice is the measurement of the activation of TcR-transgenic naïve T cells, this is not possible in other species where various surrogate readouts are used including antigen-specific re-activation of antigen-experienced T-cell clones or polyclonal T-cell lines or even proliferation of allogeneic T cells. It is known that significant differences exist in mice in the signals required for naïve T-cell priming, antigen-experienced T-cell re-activation, or allogeneic T-cell proliferation induction. Therefore, the same exact function is not fairly tested in different species. Furthermore, in species outside mice and humans, the use of epitopic peptide control requires to have accurate MHC typing and knowledge of the corresponding optimal peptides, which are generally unavailable. In addition, for accessibility reasons, the DC subsets used generally derived from different anatomic compartments depending on the species. For example, spleen DC subsets are often used in mice, blood, or tonsil DC in humans and lymph DC in sheep, which can further confound rigorous interpretation of the results when differences are observed between species. Finally, while inbred mice with defined sanitary status are generally used to limit the variability of the responses between individuals, this is not the case for other species including humans where the considerable heterogeneity in the genotypes, environments, and immune histories of individuals contribute to the strong variability of their responses (80). Hence, even for mouse experiments, there is a strong need for standardization of functional assays assessing the ability of DC subsets to process and present antigens and to functionally polarize T cells. Moreover, when attempting to compare DC subset functional specialization across two species, efforts should be made to use comparable experimental designs in both species. Thus, while functional characterization is highly desirable when identifying a potentially new subset of DC or studying in a novel context a potentially known DC subset, the identity of DC subsets must first be studied through alternative approaches measuring cell type-specific parameters that are less strongly influenced by the tissue microenvironment and the genetic or immune history of populations, and for which experimental protocols are relatively well standardized.

### Molecular profiling at the population level

**Figure 3:**
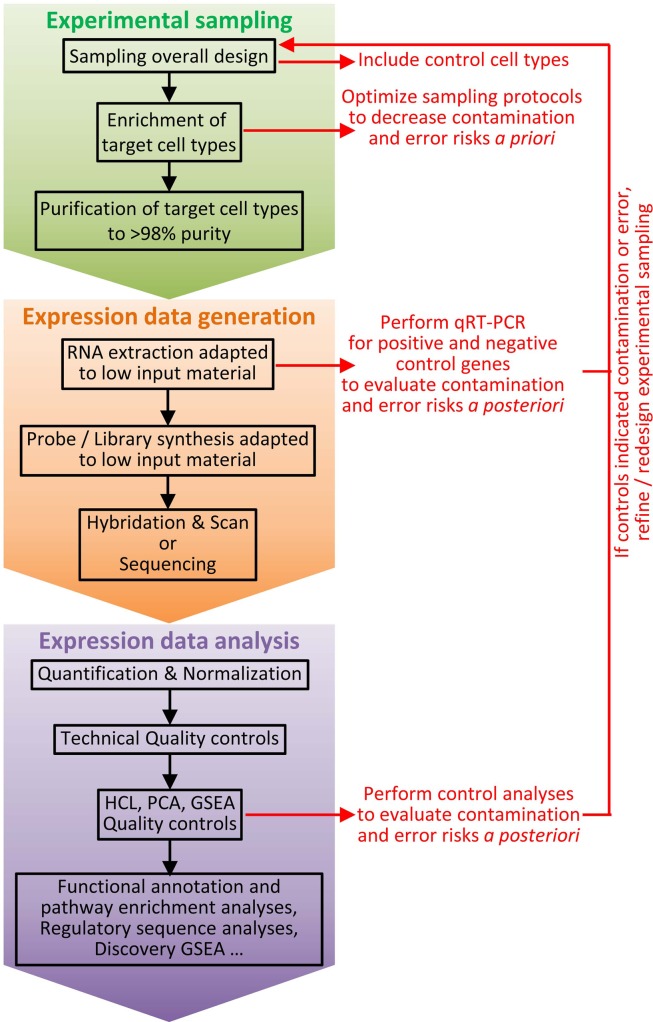
**Workflow for cell type identification by molecular profiling at the population level**. Molecular profiling at the population level can be very informative for cell type identification. However, inappropriate interpretation can occur if confounding factors are not taken into account. Hence, it is critical to carefully design experiments and to establish a rigorous workflow, including a number of key control samples and quality check procedures. The experimental sampling protocol must be optimized to decrease *a priori* the risk of cross-contamination between cell types or of error resulting in selection of another cell type than the one wanted. Purity of cell types must be assessed immediately after sampling (e.g., by flow cytometry). Positive and negative cell type controls must be included, such as sister cell types and potential contaminant populations. Once molecular expression data have been obtained, after technical quality has been validated by classical controls, additional specific quality controls must be performed to *a posteriori* ensure of lack of cross-contamination between cell subsets or to evaluate the risk of misinterpretation of the results. HCL, hierarchical clustering; PCA, principal component analysis; GSEA, Gene Set Enrichment Analysis.

As the ontogeny and functions of cell types are instructed by specific gene expression modules, cell type identity can be defined by its molecular fingerprinting, including through gene expression profiling (81, 82). Homologous cell types between species can be defined as “those cells that evolved from the same precursor cell type in the last common ancestor” (82). This implies that homologous cell types must exhibit closer molecular fingerprints and gene expression programs than non-homologous cell types. Thus, it should be possible to decipher the identity of immune cell types of virtually all vertebrate species, by establishing their gene signatures and comparing them to the transcriptomic fingerprints of the well-characterized immune cell types of the mouse referent species. This is indeed an approach we pioneered to compare mouse spleen and human blood DC subsets (39) and later extended to comparison with sheep lymph cDC subsets (76), mouse DC subsets across tissues (40), as well as chicken spleen and pig skin mononuclear phagocyte subsets (83, 84). This approach allowed us to rigorously demonstrate for the first time to the best of our knowledge that human CD1c^+^ cDC and CD141^+^ cDC were homologous to mouse CD11b^+^ cDC and CD8α^+^ cDC, respectively (39, 85). This was later confirmed by us and others based on phenotypic, functional, and ontogeny studies (18, 37, 50, 65, 86). In addition, this approach permitted to show that cDC split into XCR1^+^ and XCR1^−^ subsets in migrating skin lymph DC in sheep, a species belonging to the Laurasiatherians, which is a mammalian order distant from the mouse and human Euarchontoglires (76). This approach also provided the first compelling evidence for existence of *bona fide* cDC and macrophages in chicken, showing that diversification in mononuclear phagocyte cell types appeared in a common ancestor to mammals and reptiles (83). Comparative transcriptomics also led to recognize CADM1 and SIRPα as surface molecules whose conserved expression throughout distant species can be used as a first phenotyping step to identify XCR1^+^ and XCR1^−^ cDC subsets in any mammal (76). Notably, CADM1 is a highly conserved molecule, presenting about 90% identity across mammalian orthologs, thus allowing using commercial anti-human CADM1 antibodies for cellular staining in distant species (76, 84). We found the *Xcr1* gene among genes specifically expressed in mouse spleen CD8α^+^ DC when compared to a number of other immune cell types [see Supplementary Material “Additional file 5; gb-2008-9-1-r17-s5.xls” from Robbins et al. (39), specifically in the “CD8a_DC_gene_signature” established from our microarray data and confirmed from our own re-analysis of the microarray dataset independently generated by Dudziak et al. (87)]. Specific expression of the Xcr1 protein on mouse lymphoid tissue-resident CD8α^+^ DC and its functions were first unveiled in the pioneering report from the group of Kroczek (36), who showed that CD8^+^ T-cell cross-priming depends on their ability to secrete the Xcr1 ligand Xcl1 in experimental models where either the OVA coupled to an anti-CD205 Ab or OVA-expressing allogeneic pre-B cells are administrated *in vivo*. Xcr1 expression on CD8α^+^ DCs was also found to be critical for the optimal induction of CD8^+^ T-cell responses upon *Listeria monocytogenes* infection (18). Importantly, comparative transcriptomics revealed XCR1 as a specific and universal marker for XCR1^+^ cDC across tissues and species. This was initially shown in human, mice, and sheep (18, 37, 76) and subsequently in non-human primates and pigs (18, 37, 38, 40, 52, 59, 60). Altogether, these studies were critical for the current proposal of cDC subset classification into XCR1^+^ and XCR1^−^ cDC (38, 40). Many other recent studies have demonstrated the power of gene expression profiling to determine with a high degree of certainty the identity of mononuclear phagocyte subsets in a tissue where they had not been rigorously studied before or to identify homologous subsets of mononuclear phagocytes across species (5, 6, 8, 9, 50, 88–90). Importantly, standardized protocols for generation and analysis of gene expression data are routinely performed in many laboratories, platforms, or commercial companies in many countries. The corresponding costs have strongly decreased over the last decade and continue to go down. Hence, gene expression profiling at the population level is a very robust and reproducible methodology that is feasible in virtually all species where tools are available or can be developed to phenotypically identify and purify candidate cell subsets. However, potentially confounding factors must be taken into account to design experiments in order to reduce the risk of inappropriate interpretation of results (Figure 3). First and foremost, great care and rigor must be exerted in designing the experimental sampling protocol for cell subset purification, inasmuch as minor contamination by another cell type can dramatically impact the gene expression profile obtained. Hence, it is critical to carefully design the marker combination used to purify the different cell populations to be studied, and to control cell purity prior to the generation of the gene expression data. Second, to allow proper analysis of the gene expression profiles of the targeted cell type, appropriate cell type controls must be included, encompassing sister cell types as well as cell types that could be potential contaminants due to their expression of several of the markers used for positive selection of the targeted cell type. These controls are critical to allow assessing the risk of contamination by another cell type (49).

### Molecular profiling at the single cell level

Recent technological advances now allow performing high throughput RNA sequencing at single cell levels with high sensitivity and processivity. Transcriptomic analyses at the single cell level could solve most of the issues raised in the previous section for molecular profiling at the population level. Indeed, because it alleviates the necessity to purify cells on imperfect and potentially confounding phenotypic marker combinations, analysis at the single cell level should allow unbiased identification of potentially all cell types and their associated transcriptomic signatures. It also solves the issue of cross-contamination between cell types, since the identity of each single cell is established *a posteriori* based on the analysis of its gene expression program. In addition, the generation of gene expression data for many individual cells of the same type should increase statistical power to define genes co-expressed at the single cell level and to define cell type-specific transcriptomic modules (81). As a proof-of-principle, single cell gene expression profiling recently allowed the unbiased and *de novo* identification of the different cell types of spleen (91) and central nervous system (92, 93) via the description of their molecular identity, starting from the bulk population of all the cells that could be extracted from the organ without any prior enrichment procedure. However, molecular profiling at the single cell level cannot be used without prior phenotype-based enrichment for very rare cell types, and it is difficult to apply to species in which genome has not yet been completely assembled. To obtain complete information, including on functionally important genes for which few mRNA are expressed per cell, it is necessary to sequence at a sufficient depth of about one million reads per cell, which today still represents a very high cost when multiplied by the number of individual cells and conditions. This is all the more the case since, likewise for molecular profiling at population level, correct interpretation of the data requires that sister cell types as well as cell types that could be potential contaminants are included in the experimental design. Moreover, the technology for single cell RNA sequencing is not yet democratized, since it is challenging both for sample preparation and for data analysis. For standardization of high quality sample preparation, commercial solutions exist but are very expensive. For data analysis, there is no consensus yet on how the data should be mathematically modeled for adequate removal of background signal and for discrimination of false negative signal due to sampling bias in the pool of the cell mRNA as opposed to true lack of gene expression. In addition, the interpretation of the RNA-seq data on single cells is still largely based on the transcriptomic/molecular identity of cell types that are deduced from microarray analysis of purified cell pools (91). Hence, molecular profiling at the population level currently represents a more sustainable strategy for most laboratories.

## Recent Advances Brought by Comparative Transcriptomics at the Population Level for Defining the Identity and Functions of Mononuclear Phagocyte Subsets and Their Molecular Regulation

In this section, we will review major advances brought forward by comparative transcriptomics at the population level for defining the identity and functions of mononuclear phagocyte subsets and their molecular regulation.

Gene expression profiling of cell types with apparent ambiguous phenotype or functions allowed to rigorously establish their identity, which could be achieved properly strictly contingent to their comparison with all candidate sister cell subsets as well as more distantly related cell types. Hence, we and others showed that human blood Lineage^-^CD16^+^ cells are non-classical monocytes (39, 88) and not DC as was sometimes claimed (94–96). Similarly, analysis of human skin CD14^+^ cell expression of the transcriptomic fingerprints independently established for cDC, monocytes, and macrophages provided critical evidence that these cells are monocyte-derived macrophages (5) while they were previously designated as DC (4). Transcriptomic analyses were also instrumental to demonstrate the homology of this human dermal cell type with the murine CD11b^+^Ly6C^−^CD64^lo–hi^ (6) and pig CD163^+^ (84) skin subsets. We were also able to show that cell populations claimed to correspond to novel cell types actually corresponded to a distinct differentiation or activation state of an already known cell type, for example establishing that the so-called interferon killer DC correspond to a particular activation state of NK cells (39). Furthermore, we showed that, upon many types of *in vivo* or *in vitro* stimulation, human and murine pDC and cDC undergo a remodeling of their gene expression program related to their plasticity, including induction of NFκB and IFN target genes, but still keep the canonical gene expression associated to their subset identity (41, 97). In particular, contrary to what other researchers hypothesized (98), gene expression profiling showed that activated pDC are not undergoing a cell fate conversion into a novel type of cDC (97).

Gene expression profiling also allowed aligning subsets of mononuclear cells across tissues (6, 8, 9, 40, 55, 99), establishing cell type homologies across species (5, 39, 50, 76, 83–85, 88, 89, 100), and rigorously examining the proximity of *in vitro*-derived subsets of mononuclear cells with those naturally existing *in vivo* (39, 41, 66, 101). These studies allowed significantly advancing the ontogeny and functional characterization of mononuclear phagocyte subsets based on the novel hypotheses that can be inferred from the analysis of the gene expression programs of the cells and from their comparison with other well-characterized cell types.

The study of the functional specialization of human DC subsets was strongly boosted by the demonstration of their transcriptomic homologies with mouse DC subsets (39, 85) which was recognized as a major breakthrough in the field (37, 53, 102–104) and acknowledged to have been impossible to draw from studies based on a limited set of molecular markers (105). In particular, this led to test whether human XCR1^+^ cDC could be more efficient for cross-presentation than other human DC subsets. Even though the extent to which human XCR1^+^ cDC are more efficient for cross-presentation than other human DC subsets is debated, the results from the functional studies performed independently by many teams concurrently demonstrate that these cells excel at cross-presentation of cell-associated antigens (18, 19, 37, 41, 86, 106) and of particulate antigens delivered through FcγR, through late endosomal targeting (21, 107) or upon polyI:C stimulation (18, 41, 86, 108). In addition, in sheep, the skin lymph migrating XCR1^+^ cDC spontaneously displayed a higher efficiency of soluble antigen-presentation to specific CD8^+^ T cells, as compared to XCR1^−^ cDC (76).

Based on the demonstration of the striking transcriptomic similarities between mouse and human subsets of mononuclear cells, and on knowledge on the ontogeny of these cells in the mouse (109, 110), we proposed that, similar to their mouse counterparts, human pDC and cDC constitute a specific family of cells within the hematopoietic tree, should derive from a common progenitor with a DC-restricted differentiation potential, and could be derived *in vitro* from human CD34^+^ progenitor cells in part under the instruction of the FLT3-L growth factor (39, 85), all of which was later confirmed experimentally (41, 65, 66, 69, 111, 112).

Very importantly, comparative genomics of immune cell subsets yielded conserved transcriptomic fingerprints for each of these cell types (39), a novel knowledge which considerably accelerated the deciphering of the molecular mechanisms regulating the development and functions of leukocytes as reviewed in Table 2 (18, 36, 59, 100, 113–127). Finally, this approach uniquely allowed identifying conserved and biologically relevant cell surface markers for each subset of mononuclear cells which could enable considerably simplifying the nomenclature for DC subsets by using the same name and similar marker combinations to identify homologous cell types irrespective of their tissues and species of origin (55–57).

**Table 2:** **Genes which selective expression pattern in immune cell types was uncovered through comparative genomics and which functions in these cells were deciphered later**.

Transcriptomic signature^a^	Gene symbol (alias)	Function
pDC	*PACSIN1*	Necessary for pDC production of type I interferons upon TLR7/9 stimulation (115)
	*RUNX2*	Necessary for terminal differentiation of pDC in, and their egress from, bone marrow (114)
	*TCF4* (*E2-2*)	Master transcription factor instructing pDC development and functions (113)
	*BCL11A*	Necessary for pDC development (116, 117)
cDC	*ZBTB46* (*BTBD4*)	Transcription factor that appears to be a specific marker of the cDC and endothelial lineages and which limits spontaneous cDC maturation (118, 119, 128)
	*BATF3* (*9130211I03Rik*)	Transcription factor which can be critical for development of XCR1^+^ cDC depending on the context (121)
cDC above pDC	*BCL6*	Promotes the development of XCR1^+^ cDC (99, 120)
XCR1^+^ cDC above XCR1^−^ cDC and pDC	*TLR3*	TLR3 triggering induces a very strong activation of mouse and human XCR1^+^ cDC including a uniquely high production of IFN-β and type III IFN (41, 100, 129, 130)
	*RAB11A*	Functionally promotes cross-presentation by storing MHC class I in a unique endosomal recycling compartment (122)
Mouse XCR1^+^ cDC	*XCR1*	Likely promotes efficient interactions between XCR1^+^ cDC and NK cells or CD8^+^ T cells (18, 36)
Pan-T cells	*THEMIS* (*E430004N04Rik*)	Sets the signal threshold for positive and negative selection of developing T cells in the thymus (124–127)
	*BCL11B*	Regulates critical aspects of the development, functions, and homeostasis of T cells (123)

*^a^Transcriptomic signatures conserved between mouse and human unless specified otherwise, first reported in Robbins et al. (39), and encompassing the genes listed in this table*.

## Conclusion and Perspectives

While it might be the case in the future for single cell RNA-seq, currently no single method is sufficient to allow the best possible classification of DC. Hence, ideally, all available methods (cell surface phenotyping, gene expression profiling, functional analyses, and ontogeny) should be combined together to define DC subset identity. However, such a combination of approaches cannot be used to define cell subsets in many instances due to technical, financial, or ethical limitations. Taking these limitations into consideration, the data reviewed here show that comparative transcriptomics at the population level is currently the most robust and feasible way to define the identity of cell types. Indeed, because the ontogeny and functions of cell types are instructed by specific gene expression modules, cell type identity can be defined in a universal and unbiased way by its molecular fingerprinting, including through gene expression profiling (81). However, due to its dependency on pre-selection of cell populations based on their expression patterns of a few cell surface molecules, gene expression profiling at the cell population level is imperfect and may require iterative steps of refined cell type isolation and gene expression profiling as illustrated in Figure 3. Hence, it is all the more important that each step of the procedure is performed and rigorously quality controlled according to the best standards in the field.

Cell purity is fundamental. It is important to design a sampling method specific for each study, through identification of the most robust criteria available in the current state of the art for purification of the target cell type based on phenotypic, morphologic, or anatomical characteristics. Cell enrichment is necessary for rare cell types among bulk populations. It relies on the depletion of other populations (MACS or EasySep™ for instance). The marker combination for negative selection must not unwillingly remove a population of interest. For instance, some antibody cocktails for human DC enrichment use anti-CD16 monoclonal antibodies, so as to deplete NK cells, but this should be proscribed for the study of non-classical, CD16^+^ monocytes. Positive selection by magnetic or flow cytometry sorting is most often required after cell enrichment. Antibody labeling must be clear-cut, with separate peaks and/or selection of the events with the highest labeling and the lowest potential contamination by other populations. This selection implies the use of marker combinations specific for the population of interest, since specific markers are rarely available. XCR1 is a rare instance of a conserved marker so far only expressed on a discrete DC population. To the best of our knowledge, reliable commercial reagent are available for XCR1 staining only for mouse and rat, but XCR1 staining can also be achieved with fluorescently labeled recombinant XCL1 (40, 41, 52), a strategy that is amenable to many species in which XCL1 sequence is known. CLEC4C alias BDCA2 and LILRA4 alias ILT7 are specific markers for human pDC, but their engagement induces inhibitory signals which for instance reduce pDC production of type I interferons after stimulation (43, 77–79, 131). Although selectively expressed at high levels on human pDC in the blood or lymphoid organs under steady-state conditions, NRP1 alias BDCA4 can be induced on activated cDC and is also expressed on other cell types including neurons, endothelial cells, and tumor cells (132, 133). CD123 is a good marker to help identifying pDC in non-human primates, but it also labels mastocytes which are present in the blood or in lymphoid organs (134). Cell purity must be controlled in each experiment, by flow cytometry re-analysis just after sorting, and as one of the first step of transcriptomic analysis by examining the expression of negative and positive control genes (expression of genes that should be expressed only on other populations including potential contaminants, and expression of genes characteristic for the population of interest including but not restricted to genes coding for the molecules used for positive selection) (Figure 3).

The quality and quantity of mRNA must be adequate, even when cell numbers are low. RNA extraction kits adapted to low cell number samples may be required. mRNA quality must be controlled by electrophoresis. A linear amplification protocol must be used, that has been validated for yielding results from low input RNA showing a strong correlation with the results obtained with higher RNA input and a classical amplification procedure.

For bioinformatics analyses, the dataset must include sister cell types as well as the cell types the most likely to contaminate the cell type of interest, or at least be compatible for integrative analysis with a reference dataset including these control populations. Several independent methods for data analysis should be used, to ensure robustness of interpretation. Beyond relative classification of the cell types of the dataset by classical approaches computing the overall distance between their gene expression programs as performed by hierarchical clustering or principal component analysis, the identity of cell types can also be reliably inferred from the analysis of their relative expression of robust cell type-specific gene signatures established from re-analysis of public gene chip databases and/or from published articles.

Novel advances are being brought through molecular profiling of subsets of mononuclear cells. In addition to steady-state conditions, populations can be analyzed after stimulation to identify the specific activation pathways elicited in pure cell populations or upon interaction between different cell types (41, 97, 135–137). In addition to unbiased analysis of the cellular composition of different organs (91, 93), transcriptomic profiling at the single cell level will allow studying heterogeneity in gene expression within one cell type with the hope to link it to functional heterogeneity (138) and eventually with the former history/epigenetic imprinting of each cell. Comparative transcriptomic studies allowed us and others to identify in humans, non-human primates, pig, sheep, and chicken cDC subsets homologous to those well described in mice (5, 18, 39, 50, 52, 62, 76, 83–85). These studies suggest that similar cDC subsets already existed in the last common ancestor of birds and mammals. Conserved gene modules appear during evolution to elicit new functions (81, 82). For instance, regarding T helper lineage diversification during evolution, contrary to bony fishes, the elephant shark, a cartilaginous fish, has been reported to lack genes encoding for critical transcription factors or cytokines instructing the development or involved in the functions of Th2, Th17, and Treg cells, such as RORC and FOXP3, IL-4, IL-21, IL-23, and IL-2 (139). This suggests that the genes required for the development of the different T helper lineages might have appeared progressively as modules during evolution starting in bony fishes and with late development of the Treg and Th17 lineages (81). Comparative genomics of mononuclear phagocyte subsets and single cell gene expression profiling will critically help identifying novel gene modules and their associated immune functions. In pDC, evolutionarily conserved co-expression of *TCF4*, *RUNX2*, *TLR7*, *TLR9*, *UNC93B1*, *MYD88*, *IRAK4*, *IRF7*, and *PACSIN1* might represent part of a gene module instructing the functional specialization of this cell type in high level production of type I interferon in response to sensing of oligonucleotide sequences of viral or autologous origin. In XCR1^+^ cDC, evolutionarily conserved co-expression of *CLEC9A*, *SYK*, *RAB11A*, *RAB7B*, *SEPT3*, *SNX22*, *TLR3*, *CADM1*, and *XCR1* might represent part of a gene module instructing the functional specialization of this cell type in CD8^+^ T-cell activation and specifically in cross-presentation of cell-associated antigens. In any case, the discovery of the sets of genes that are tightly co-expressed in DC subsets across various tissues and species, not only at the population level but also at the single cell level, should allow identifying the gene modules instructing DC subset functions. Characterization of the members of these gene modules which role in DC is unknown yet should strongly contribute to increase our knowledge on DC subset functional specialization and their molecular regulation. Of note, not all of these gene modules might harbor the same differential pattern of expression between DC subsets in different animal species. Some functions have gained or lost expression in specific cell subsets in some species which should correlate with similar changes in the expression patterns of the corresponding gene modules. For instance, IL-12 is produced both by pDC and cDC in mice, but only by cDC in humans, while antigen cross-presentation appears to be more strongly associated with XCR1^+^ cDC in mice than in humans (18, 19, 22). Isolation and comparison of mononuclear phagocyte subsets from homologous organs in different species may help understand how the anatomical compartmentalization of these cells is established and affects their functions, including local interaction with specific cell types and chemokines. Dating when during evolution pDC as well as classical and non-classical monocyte subsets appeared, and in which anatomical compartments they reside in the species the most distant to humans and mice, may give novel insights into the core functions of these populations.

*In vivo* manipulation of DC can promote and orient immune responses based on the intrinsic functional properties of the DC subset targeted and can be advantageously used for prophylactic vaccination or immunotherapy against cancer or infections. This strategy can benefit from the knowledge gained from the expression profiling of DC subsets and their alignment across species. Notably, based on their homology with mouse XCR1^+^ cDC, human XCR1^+^ cDC can be considered as a promising target when cross-presentation is desirable, in particular for fighting cancer or infections by intracellular pathogens (23, 24, 29, 72, 73, 140–143). Moreover, because it is specifically expressed in XCR1^+^ cDC in a conserved manner in evolution, and it has been successfully used for *in vivo* delivery of antigens specifically to XCR1^+^ cDC to vaccinate mice (23, 24), XCR1 can be considered for a universal DC targeting strategy in potentially all vertebrate species. Interestingly, the targeting of XCR1 can be achieved with targeting units composed of recombinant XCL1 fused to protective antigens in the form of vaccibodies (24), a strategy that is amenable to many species in which the XCL1 sequence is known. Although more broadly expressed in the DC lineage at least in mice, CLEC9A is also an interesting target since it directly promotes cross-presentation of the material it binds, probably by delivering it into appropriate endosomes (144, 145), and because it is selectively expressed to high levels on XCR1^+^ cDC in humans, sheep, and mice (25, 32, 76, 146) although it may not be the case in some other species such as pig. Arguments in favor or against the targeting of XCR1^+^ cDC in the clinic are summarized in Table 3. The identification of XCR1^+^ cDC in companion and sport animals, and in animals of the agro-economy, such as ruminants, pigs, poultry, and fishes, will allow designing better vaccines to protect them against infections in order to ameliorate animal welfare and to prevent pandemics causing severe economic losses. It will also contribute to a global public health strategy because some of these animal species as well as wild animals are targets or reservoirs for major zoonotic pathogens. The identification of XCR1^+^ cDC in rhesus macaques and in pigs opens the way to preclinical vaccination studies in these species which are close to humans. Vaccibodies based on XCL1 dimers coupled to influenza or SIV proteins are planned to be used for vaccination of pigs or rhesus macaques, respectively, and induction of immune responses and protection against infection. pDC targeting could also be considered as an interesting alternative for vaccination against viruses or tumors (20, 147, 148), or for the induction of cross-tolerance to treat autoimmune diseases or food allergies (149, 150).

**Table 3:** **The PROs and CONs for *in vivo* targeting of XCR1^+^ cDC^a^**.

	PROs	CONs
Cross-presentation efficiency	Higher for blood and skin XCR1^+^ cDC, especially for cell-associated antigens	Disputed for XCR1^+^ cDC from secondary lymphoid organs (19, 22) depending on intracellular compartment of antigen delivery (21)
Anatomical localization	Present in lymphoid and non-lymphoid tissues, enabling subcutaneous, intradermal, or oral vaccination	Low efficiency of human XCR1^+^ cDC for induction of mucosa-homing CD8^+^ T cells (151)?
Frequency	Few cells can mediate important functions *in vivo*. Quality matters more than quantity	Very few numbers of XCR1^+^ cDC in most tissues
Specificity of targeting	Very specific expression of XCR1 as opposed to the broader expression of CD141, DEC205, and CLEC9A. Precise targeting and better pharmacodynamics	Too specific, limiting biological effect to just one DC subset, may not induce strong enough or broad enough immune responses
Responsiveness to adjuvants	Very good responsiveness to PolyI:C. PolyI:C is a very potent adjuvant for the induction of strong, polyfunctional CD8^+^ T-cell responses which might result in part from TLR3 triggering in XCR1^+^ cDC	PolyI:C may primarily work by activating other targets, i.e., non-immune cells expressing TLR3 or cells activated through MDA5
Proof of concept achieved in mice	XCR1^+^ cDC are critical for anti-tumoral responses in mice (72, 121, 152, 153). XCR1 targeting works in mice (23, 24). XCR1 bio-equivalency in human, macaques, mouse, pig, and sheep, same gene expression pattern and biological function. Hence, higher probability of translation to human of mechanistic studies in animals	Many previous failures of mouse to human translation
*In vitro* model	Ability to generate *in vitro* and manipulate *bona fide* human XCR1^+^ cDC from CD34^+^ cord blood progenitors (41, 65, 66, 69, 111, 112)	
Cytokine production	XCR1^+^ cDC can produce IL-12 but maybe optimal conditions to induce this function remain to be identified (50, 65, 66, 143). Mouse and human XCR1^+^ cDC are high producers of beta and type III interferons upon PolyI:C stimulation (41, 100, 129, 130)	Human XCR1^+^ cDC are very poor producers of IL-12 (70, 108)
Clinical data	Gene expression profiling of human tumors suggest that infiltration by XCR1^+^ cDC but not other myeloid cells is of good prognosis both in mice and humans (72)	Formal measurements of XCR1^+^ cDC infiltration in human tumors and of its beneficial role for disease control remain to be established

*^a^More details and bibliographical references can be found in the main text of this review*.

A synthetic list of phenotypic, transcriptomic, and functional hallmarks which have already allowed conserved identification of different DC and monocyte subsets in humans and mice is presented in Table 4. The present Special Issue and future workshop on DC nomenclature will help reach a consensus panel for practical definition of the populations, in order to integrate faster the huge, but scattered knowledge accumulated by different laboratories in different cell types, species, and organs.

**Table 4:** **Practical guidelines for consistent definition of DC subsets across mouse and human tissues with potential applicability to other mammals**.

Characterization	XCR1^−^cDC2		XCR1^+^ cDC1		pDC	
	High or positive	Negative or low	High or positive	Negative or low	High or positive	Negative or low
Conserved phenotype	CD11c^high^	CD3^−^	CD11c^low-to-high^	CD3^−^	MHC-II^int^	CD3^−^
	MHC-II^high^	CD19^−^	MHC-II^high^	CD19^−^	FLT3^+^	CD19^−^
	FLT3^+^	CD14^−/low^	FLT3^+^	CD14^−/low^		CD14^−/low^
	SIRPα^+^	CD206^−/low^	XCR1^+^	CD206^−/low^		CD206^−/low^
		CD123^−^	CADM1^+^	CD123^−^		CD19^−^
Critical species-specific phenotypic markers					Mouse: Siglec-H or Ccr9	
					Human: CD123 and CLEC4C (BDCA2) or ILT7 (LILRA4)	
Hallmark genes (18, 37, 39)	*FLT3*	*XCR1*	*FLT3*	*TLR4*	*FLT3*	*XCR1*
	*TLR8*	*RAB7B*	*XCR1*	*TLR7*	*TLR7*	*CADM1*
	*ZBTB46*	*GCET2*	*CADM1*	*IRF4*	*TLR9*	*TLR3*
	***IRF4****^a^*	*TLR4*	*TLR3*	*TCF4*	*PACSIN1*	*TLR8*
		*IRF8*	*RAB7B*	*RUNX2*	***IRF8***	*RAB7B*
		*TCF4*	*GCET2*	*SPIB*	***TCF4***	*GCET2*
		*RUNX2*	*ZBTB46*		***RUNX2***	*ZBTB46*
		*SPIB*	***IRF8***		***SPIB***	*BATF3*
			***BATF3***		***BCL11A***	*CADM1*
Hallmark cytokine production	IL-23 production? (62)		Type III interferon production upon TLR3 triggering (41, 100, 129, 130)		Production of type I and III interferons in response to TLR7/9 triggering	
Hallmark antigen-presentation functions	High efficiency for CD4^+^ T-cell activation		High efficiency for CD8^+^ T-cell activation, in particular through cross-presentation of cell-associated antigens			

*^a^Master transcription factors critical for cell subset development are indicated in bold font*.

## Conflict of Interest Statement

The authors declare that the research was conducted in the absence of any commercial or financial relationships that could be construed as a potential conflict of interest.
